# Perceptions and attitudes of patients and healthcare workers towards the use of telemedicine in Botswana: An exploratory study

**DOI:** 10.1371/journal.pone.0281754

**Published:** 2023-02-16

**Authors:** Benson Ncube, Maurice Mars, Richard E. Scott

**Affiliations:** 1 Department of TeleHealth, School of Nursing & Public Health, College of Health Sciences, University of KwaZulu-Natal, Durban, South Africa; 2 Dynamics Research & Development Institute, Gaborone, Botswana; 3 College of Nursing and Health Sciences, Flinders University, Adelaide, South Australia, Australia; 4 Department of Community Health Sciences, Cumming School of Medicine, University of Calgary, Calgary, Alberta, Canada; Universita degli Studi di Milano, ITALY

## Abstract

**Introduction:**

In March 2020, the Botswana Ministry of Health and Wellness approved a National eHealth Strategy. Although a milestone, the strategy does not mention telemedicine. There is need to address this by developing an evidence-based adjunct strategy for telemedicine to facilitate its introduction and adoption. To do so, several stages of a published eHealth Strategy Development Framework were mimicked. This allowed situational awareness to be created through exploring behavioural factors and perceptions that might influence the adoption of telemedicine in Botswana. The study aim was to explore current issues, concerns, perceptions, attitudes, views, and knowledge of patients and healthcare professionals regarding health-related issues and telemedicine that might influence implementation of telemedicine in Botswana and thereby inform future development of a telemedicine strategy.

**Methods:**

An exploratory survey study was conducted using different survey questionnaires for patients and healthcare professionals, each using a mix of open- and closed-ended questions. These questionnaires were administered to convenience samples of healthcare professionals and patients at 12 public healthcare facilities in Botswana; seven clinics (three rural; four urban), and five hospitals (two primary, two district, and one tertiary), selected to align with the country’s decentralised healthcare structure.

**Results:**

Fifty-three healthcare professionals and 89 patients participated. Few healthcare professionals had actively used telemedicine for clinical consults and self-education using telephone calls, cell phone apps, or video conferencing (doctors 42%, nurses 10%). Only a few health facilities had telemedicine installations. Healthcare professional preference for future telemedicine uses were e-learning (98%), clinical services (92%), and health informatics (electronic records (87%). All healthcare professionals (100%) and most patients (94%) were willing to use and participate in telemedicine programmes. Open-ended responses showed additional perspective. Resource shortages (health human resources and infrastructure) were key to both groups. Convenience, cost effectiveness, and increased remote patient access to specialists were identified as enablers to telemedicine use. However inhibitors were cultural and traditional beliefs, although privacy, security and confidentiality were also identified. Results were consistent with findings from other developing countries.

**Conclusion:**

Although use, knowledge, and awareness of telemedicine are low, general acceptance, willingness to use, and understanding of benefits are high. These findings bode well for development of a telemedicine-specific strategy for Botswana, complementary to the National eHealth Strategy, to guide more systematic adoption and application of telemedicine in the future.

## Introduction

Africa, more so sub-Saharan Africa, has an acknowledged and marked shortage of healthcare professionals, an excessive burden of disease, and poorly funded healthcare provision [1–4]. To combat some of these concerns and issues, use of innovative interventions such as eHealth / digital health [4] is recommended (including telemedicine–"The delivery of health care services, where distance is a critical factor, by all health-care professionals using information and communications technologies…") [2]. Similarly, development of an eHealth / digital health strategy by each country is recommended [5, 6]. Indeed, Maina and Singh recently argued that “eHealth strategies have become mainstream” and guide intervention [7]. However, this global endeavour to implement eHealth has not yet yielded the desired outcomes.

Except for the concept of technology leapfrogging, the evolution and growth of telemedicine within the developing world mirrors that of the developed world a decade or more ago, with typically slow, often ad hoc, and unplanned development, plus musing about the value of national strategy [8]. Thus, telemedicine uptake in sub-Saharan Africa has also been slow [1], despite many countries having some form of information and communication technologies (ICT), health information system, or even eHealth strategy [9]. A potential explanation for this dichotomy may be that if telemedicine is not an embedded focus of an existing strategy, insufficient attention will be given to its adoption and implementation. In contrast, some developed countries have embedded telemedicine into their eHealth strategies and implemented specific telemedicine action plans, calling telemedicine the ‘key to health services of the future’, thereby improving their nationwide application of telemedicine [10]. Given COVID-19, the US now considers telemedicine a ‘low resource’ option whose use will be maintained, perhaps increase, post-pandemic [11]. Botswana developed its eHealth strategy while concomitantly trialling several mHealth initiatives with little success. Yet few sustained telemedicine initiatives or benefits have been realised from this investment [12], perhaps because of ‘poor strategy development’ or the absence of a specific telemedicine strategy.

The Government of Botswana, through the Ministry of Health and Wellness, provides free healthcare services to its citizens but as a fee for service to foreign nationals. The healthcare system is well structured, highly decentralised, and provides primary care through a hierarchical network of health facilities (mobile posts, health posts, clinics, primary hospitals, district hospitals, and tertiary hospitals). These health facilities are grouped into 27 health districts and include some private health facilities [13]. Similar to many countries, Botswana has a maldistribution of healthcare workers [14], being relatively concentrated in the largest and only city with a population over 100,000, Gaborone (~ 208,500).

Studies have shown a marked burden of both communicable and non-communicable diseases (NCD) in sub-Saharan Africa (SSA), with a surge in NCDs in the last two decades which are expected to exceed communicable, maternal, neonatal, and nutritional diseases (combined) as the leading cause of mortality by 2030 [15, 16]. Exacerbating this is the shortage of healthcare workers; 0.8 workers per 1,000 population across Africa, with just 9 countries in SSA with a health workforce density above 2.28 per 1,000 people, the minimum MDG density threshold (Botswana at 2.7) [17, 18]. There are only 815 general practitioners and 38 specialists in Botswana to address the healthcare needs of the ~2.35 million residents [17, 19]. In perspective, the WHO reports one physician for every 3,623 Africans, compared to one for every 232 Europeans, and notes that the shortage contributes to the inequalities in health service coverage and burden of disease experienced by SSA [20], including Botswana [21].

The country’s National eHealth Strategy, many years in development, was formally approved and released on 10 March 2020 [22]. It makes no mention of telemedicine and, as a consequence, the strategy does not culminate in a distinct, evidence-based strategy and subsequent implementation plan for telemedicine in the country. This lack of focus on telemedicine is a major omission of the National eHealth Strategy that must not be overlooked. Its remedy necessitates the development of a separate but complementary telemedicine strategy for Botswana.

This need has been amplified by the recent COVID-19 pandemic, which continues to disrupt already overburdened and short-staffed healthcare delivery systems, especially those in Africa. The pandemic has inadvertently raised awareness of eHealth (in particular telemedicine), and spurred rapid adoption and new innovations to leverage the technology’s health system, healthcare, and health benefits [23]. The wider literature also suggests this renewed application and integration of telemedicine implementations will continue beyond the pandemic era [23–25]. This will necessitate the resolution of many legal, regulatory, and ethical issues that appear to be overlooked or conveniently bypassed in the desire to mitigate the risks of the current pandemic. Planned, evidence-based, and health needs-based implementation of technologically appropriate, culturally sensitive, and environmentally aware telemedicine solutions (within a legal, regulatory, and ethically sound setting) could be effectively addressed in a distinct but complementary telemedicine strategy.

However, there is limited published guidance for eHealth strategy development, with perhaps only four practical and detailed guidance documents for generic eHealth strategies available [6, 9, 26, 27]. In this current study, the strategy development framework proposed by Scott and Mars was adopted, and several of the 7 Steps outlined in the eHealth Strategy Development Framework (eHSDF) are emulated [9], focussed on situational assessment and review. Because telemedicine is a subset of eHealth, this emulation process can be used to develop a telemedicine-specific strategy that identifies needs, inhibitors and enablers as well as the preferred telemedicine solutions for Botswana. The theoretical grounding and applied evidence-based research underpinning the eHSDF, together with the clarity of its described Steps, provided the rationale for its adoption. Nonetheless for academic purposes, as is the case in this study setting, limitations exist. The level and scope of activity required at a national level could not be achieved within a study, requiring the eHSDF process to be mimicked.

Seven fundamental principles guide the eHSDF process [9]. There are also seven sequential steps. Step1 (‘Evidence Gathering and Situational Assessment’) refers to gathering, reviewing and interrogating the available information related to specific health issues of most importance. Step 2 (‘Holistic Review’) is a situational assessment, examining other confounding factors beyond health issues (e.g. social determinants of health such as socioeconomic, political, and contextual aspects). Step 3 (‘Differential Diagnosis’) applies to information and geographical disaggregation to discover any differences in identified health issues which might appear similar, yet require different solutions. Step 4 (‘Preliminary Prioritisation’) identifies the top priority health issues for further review. Step 5 (‘Identifying Solutions’) is when a range of possible solutions that could address the identified needs (both technological and non-technological) are also identified. Step 6 (‘Considering eHealth Solutions’) is the stage at which only eHealth interventions, i.e., technological solutions, are identified for consideration. Step 7, (‘Secondary Prioritisation’) is the most critical stage when the most ‘technologically appropriate’ solutions [9] are selected and prioritised for implementation, thus providing the direction for investment decisions and policy developments. Thereafter the findings from Steps 1–7 are synthesised, culminating in the eHealth strategy document.

In emulating aspects of Steps 1, 2 and 3 this study serves as a formative undertaking towards the development of an evidence-informed telemedicine strategy to complement the existing eHealth strategy for Botswana. To this end, the study used questionnaires to achieve its aims: to explore the perceptions, attitudes, views and knowledge of patients and healthcare professionals about telemedicine, its use, and the factors that might influence implementation of telemedicine in Botswana, as well as their perceptions of the current health-related issues that they face in order to inform future development of a telemedicine-specific strategy.

## Methods

An exploratory survey study was conducted, using both open-ended and closed-ended questions. Healthcare professionals and patients at 12 health facilities were surveyed using convenience sampling.

### Survey instruments

Informed by the literature the authors developed two survey questionnaires, in English; one for healthcare professionals (HCP) and one for patients. The purpose of the surveys was to explore participants’ understanding of telemedicine, their perceptions of the potential benefits and challenges to its use and factors that could influence its use. In addition, their perceptions of the most important disease issues they and their communities face, and problems with the healthcare system were investigated. The questionnaires were pre-tested by five HCP and six patients respectively, and their feedback used to improve questionnaire constructs and face validity.

The questionnaires used open-ended, dichotomous, multiple choice, and Likert scale options, and were grouped into domains. The HCP Domains were: Demographics, telemedicine awareness, telemedicine knowledge, e-readiness, telemedicine benefits, cultural influences, HCP willingness to use telemedicine, patient willingness to use telemedicine, resource shortages, and service preferences. For HCP, there were 35 primary questions for which 75 responses were possible. Of the 75 possible responses, 29 were open-ended, 20 were dichotomous, 11 were Likert, three were multiple choice, and 12 were hybrid (six multiple choice questions each with an open-ended ’other’ response option). The Patient Domains were: Demographics, telemedicine awareness, e-readiness, telemedicine benefits, cost, resource shortages, willingness to use telemedicine, health needs, disease prevalence, challenges and risks associated with telemedicine, and previous remedial measures. For patients, there were 36 primary questions for which 68 responses were possible. Of the 68 possible responses, 21 were open-ended, 13 were dichotomous, 21 were Likert, five were multiple choice, and eight were hybrid (three multiple choice questions each with an open-ended ’other’ response option; one dichotomous with an open-ended comment option). The respondents’ perceptions of current health-related issues and concerns were addressed across several of these domains.

### Study setting, locations, and sampling

Twelve healthcare facilities (three rural clinics, four urban clinics, two primary hospitals, two district hospitals and one referral hospital) within three representative health districts (Gaborone, and Kweneng East and Mahalapye; west and north-east of Gaborone), were surveyed in August 2019. The health facilities were selected to respect the hierarchical structure of the public health system and the density of HCP.

Convenience sampling was used to recruit HCP and patient participants in the outpatient departments of the facilities at the 12 sites. The inclusion criteria for participants were: health professionals were a doctor or nurse working at the facility and willing to give consent to participate in the survey; patients were over the age of 18 years, read and spoke English, and willing to give consent to participate in the study. After giving consent patients were handed a survey for completion and return. Healthcare professionals on duty at the same facilities who agreed to participate and consent were given a survey for completion and return after three days. Other HCPs were emailed a copy for online consent, for completion, and return. Open-ended questions were qualitatively analysed, while descriptive statistics (percentages and frequencies) were used to describe categorical data.

### Survey approach

Although difficult to quantify, the rationale was as follows. The populations of interest were not all clinicians nor the entire population, but rather those providing and seeking care at a given point in time. There are only 815 general practitioners and 38 specialists in Botswana to address the healthcare needs of the 2.35 million residents (2022) [17, 19]. Further, both the clinician and general populations are concentrated in urban centres, primarily Gaborone. More specifically, given the number of clinicians and patients in the selected centres (proportionate to the total number of clinicians working in, and the number of patients seeking care from, these healthcare facilities on any particular day) exploring in and around the setting of the primary population centre would yield the perspective and situational awareness required. To gather the desired insight, it was decided that a survey (with question diversity) be used, rather than a narrower approach using key informant interviews, focus groups, or even a survey of a smaller group. The survey questions were aligned with the situational awareness needs of the chosen eHealth Strategy Development Framework.

### Data analysis

Questionnaire responses were entered into an Excel spreadsheet (Microsoft Excel ^®^) by one author (BN) and quality checked by another (MM). NVivo 11 (QSR International ^®^) was used to aid thematic and content analysis of the open-ended responses. A qualitative data reduction process was applied (BN) moving from data familiarisation, to codes, to themes, and defining and naming the themes [28, 29]. Related themes were categorised and a code book generated, reviewed and evaluated by all authors for verification, and findings summarised as themes and frequencies (reported as percentages). Excel was used to conduct content analysis of open-ended questions by coding each response into a category and determining the frequency for each category [30], and for descriptive analysis (frequencies and percentages) of responses from closed-ended questions (BN, MM). Percentages were primarily used to report findings for easy interpretation. Analyses were reviewed and discussed by all authors for verification.

The Excel data spreadsheet was reviewed by all authors for insightful quotations, and reported verbatim where they added perspective. Findings for HCP and patients were summarised under four common headings: Context; health-related issues and concerns; prevailing perceptions, attitudes, views and knowledge of telemedicine; and factors influencing implementation of telemedicine.

### Ethics statement

Ethical approval for this study was granted by the Ministry of Health and Wellness of Botswana, the respective hospitals, and the Humanities and Human Sciences’ Ethics Committee of the University of KwaZulu-Natal, South Africa. All participants provided written consent.

## Results

The demographic and contextual results for both groups are presented in Table 1.

**Table 1:** Demographic characteristics and context (awareness and use of technology) of healthcare professionals and patients.

	Healthcare professionals			Patients *			
Demographics	Doctors	Nurses	Total	Employed	Unemployed	(Student)^τ^	Total
Sample size	33	20	53	67	22	14	89
	n (%)	n (%)	n (%)	n (%)	n (%)	n (%)	n (%)
Male	19 (57)	6 (30)	25 (47)	28 (42)	8 (36)	5 (36)	36 (40)
Female	14 (42)	14 (70)	28 (53)	37 (55)	14 (64)	9 (64)	51 (57)
**Total**	33 (100)	20 (100)	53 (100)	67 (100) *	22 (100)	14 (100)	89 (100)
**Use computer to do work**							
At work	30 (91)	10 (50)	40 (75)	41 (61)			58 (66)
At home	29 (88)	10 (55)	39 (74)		13 (59)	11 (79)	
**Internet connectivity**							
At work	28 (85)	12 (60)	40 (75)	41 (61)		9 (64.2)	50 (56)
At home	30 (91)	8 (40)	38 (72)	30 (45)	14 (64)	10 (71.4)	54 (61)
**Heard of TM**	28 (85)	7 (35)	35 (66)				
**Used TM**	14 (42)	2 (10)	16 (30)				
**Knew of TM introduction to some Botswana hospitals**				10 (15)	1 (4)	1 (4)	11 (12)
**Travel time (≤2h) to nearest referral hospital**				59 (88)	6 (27)	11 (79)	76 (85)
**Have visited MOHW website**				11 (16)	4 (19)	4 (29)	15 (17)

* One female patient did not disclose her employment or student status; two patients did not disclose their gender.

^τ^ Students are a subset of the unemployed.

TM = Telemedicine

The themes and sub-themes derived from qualitative analysis of the open-ended questions are presented in Table 2.

**Table 2:** Themes and sub-themes from qualitative analyses.

Healthcare Professionals (n = 53)	Patients (n = 89)
**Theme / sub-theme**	**Theme / sub-theme**
*Advantages and benefits of telemedicine*	*Advantages and benefits of telemedicine*
Provision of care	Time saving
Improved access to care	Cost saving
Facilitate second opinion and referral	Convenience
*Resource shortages in healthcare system*	Efficiency
Clinical support	Availability
Staff	*Resource shortages in healthcare system*
Poor systems / infrastructure	Doctors
Medications and equipment	Midwives
*Challenges to using telemedicine (self)*	Nurses
Lack of skills and knowledge	Transportation
Connectivity	Equipment
*Perceived challenges to using telemedicine (other HCP)*	Referral (lack of staff)
Lack of skills and knowledge	*Effect of culture on telemedicine*
Connectivity	Positive effect
*Perceived challenges to using telemedicine (patients)*	Negative effect
Confidentiality	*Public policy participation*
Poor resources and infrastructure	Consultation and contribution
	Determine community needs

### Healthcare professionals

#### Context

The survey was completed by 53 HCPs (33 doctors and 20 nurses). Their predominant age ranged from 31 to 40 years, and work experience from <5 (30%) to ≥10 (40%) years.

Telemedicine use was reported at four hospitals and one urban clinic, but only 14 doctors and 2 nurses had used telemedicine, primarily for clinical consultation in oral medicine and teleradiology, with some use for teledermatology, telepathology and cervical screening, or for education. Common modes used were telephone calls, cellphone apps, email, and videoconferencing, with very limited use of instant messaging or facsimile machines. Of those who had not used telemedicine, most believed that both patients (76%) and HCPs (100%) would be willing and able to its use it. Knowledge of telemedicine was obtained mostly from colleagues (75%) and, to a lesser extent, the Internet (38%), and scientific meetings and workshops (38%). Few were aware that Botswana had, at that time, a draft eHealth Strategy (28%).

#### Health-related issues and concerns

All but one noted shortages of resources: material (remote clinics; hospital beds; medical equipment (94%), human health personnel (93%), technology (93%), and financial (89%)), and that telemedicine could be used to overcome or address these shortages (91%). Human resource shortages of doctors (91%), nurses (70%), midwives (43%), and specialists (19%) were noted by essentially all participants.

The most pressing health related problems, challenges, or needs identified in their work environments were clinical support, equipment and staffing. Many comments reflected this: “Lack of experts in some specialties”, “Lack of basic diagnostic facilities and drugs”, “Lack of resources, e.g. medications and other medical equipment”, “Supply of medications and others medical consumables and equipment—a huge problem in Botswana”. Markedly fewer responses were seen for issues such as information management, health system efficiency, transportation, Internet access, power, or education (“Network, power failure”, “There is no electronic medical record—IPMS only contains labs”, “Internet connectivity is needed in my clinic to improve the utilisation of patient data and patient care”). The top five diseases of most concern were cancer (42%), HIV (39%), hypertension (36%), tuberculosis (33%), and diabetes (33%) for doctors, and hypertension (80%), sexually transmitted infections (70%), HIV (60%), diabetes (50%), and tuberculosis (45%) for nurses.

#### Prevailing perceptions, attitudes, views and knowledge of telemedicine

All agreed that telemedicine would facilitate second opinion and referral decisions and provide both patients and HCP access to specialists. The majority (96%) felt that it may be cost effective for rural and remote healthcare delivery. There was some agreement that patients may not like the virtual contact (59%; “Miss interaction with doctor”, “Lack of physical examination”) or be concerned about confidentiality issues (42%; “confidentiality concerns and cultural shocks”), or simply dislike for change (“Change, fear of new things”, “The acceptance by the community in remote area”). HCPs own telemedicine preferences were for e-learning (98%), clinical services (92%), and health informatics (e.g. Electronic Medical Record; surveillance) (87%).

Of note was differing perspective about the primacy of telemedicine for addressing health needs. Of physicians solely in management positions, 66% believed alternatives to telemedicine could address identified healthcare needs, compared to 47% of physicians in clinical service.

#### Factors influencing implementation of telemedicine

The main reasons for using telemedicine were projected to be for consultation (74%), seeking second opinion (55%), providing specialist care (41%), education (21%), and to reduce travel (17%). Telemedicine was identified for potential use in 36 areas of medicine, of which preference was given to radiology, pathology, education, patient consultation, and surgery as the top five health services. Although challenges for adoption and implementation of telemedicine were recognised by respondents, the majority indicated they believed they (68%), other HCPs (72%), and patients (68%) would be able to address these challenges.

HCPs identified from their own perspective, and for other HCPs, the following as challenges or barriers to telemedicine use; poor network connectivity (“The internet connection is not reliable”), lack of knowledge and skills to use telemedicine (“Availability of computers, training”), time constraints, cost (“Costs of IT equipment, WiFi coverage, steady internet”), and lack of appropriate equipment (“Access to necessary equipment”). Few HCPs (6%) identified no challenges to implementing telemedicine. In contrast HCPs identified the following as challenges or barriers that patients might experience; trust and confidentiality (15), network connectivity (11), lack of skill and understanding of telemedicine (8), and cultural beliefs of face-to-face consultation preferences (4).

Patient respondents

#### Context

The survey was completed by 89 patients. Most patients attended Government hospitals or clinics for their healthcare (88%) and lived within 2 hours travel of the closest referral hospital (85%), although the remainder indicated travel times of up to and including in excess of 8 hours. Only one unemployed patient attended a private general practice, and few employed patients attended private facilities—6% for private general practitioners and 6% for private hospitals.

The main source of information regarding telemedicine was by word of mouth (71%); other sources included television, the Internet, with minimal contribution from Government material or health meetings. Few patients had visited the Ministry of Health website (17%) which was mainly visited for information purposes and checking on health facilities. Most agreed computer literacy and spending leisure time on social networks was common in modern times (90%) and should facilitate use of telemedicine (73%). All indicated they were willing to learn more about telemedicine, and 94% to use it. Over 90% indicated they would do so with confidence if provided access to reliable and trained clinicians, and would encourage others to do so.

Some patient comments suggested a desire to be involved in the telemedicine policy development process as a matter of principle (“So that everyone gives their own view”, “Public are the people who use facilities, so the policy should be influenced by people”). Other views seemed more practically oriented (“Because people should know about the developments in their community”, “Patients are the beneficiaries of the services therefore could advise on improvements of medical services”).

#### Health-related issues and concerns

The most pressing health-related challenges or needs in patients’ communities were: staff shortages (40%), lack of transportation capability (30%), lack of equipment (30%), problematic diseases (24%), queuing challenges (12%), and lack of medical supplies (11%). The most pressing diseases were considered to be: blood pressure—high or low (92%), HIV/AIDS (82%), tuberculosis (73%), cancer (69%), and diabetes (60%). Most patients indicated they felt nothing had been done to address these issues in their communities (60%), a sentiment reflected in many curt comments—“nothing” or “nothing; still pending”. However, mention was made by between 1 and 5 participants of attempts at education, recruitment, a mobile clinic, and increased referral, although the latter was viewed with scepticism: “Referral to another facility while there are no doctors” and “Referrals with long time frames”.

Most were concerned about shortages of resources (human—91%; material (remote clinics; hospital beds; medical equipment) - 91%; or technical—76%), and believed telemedicine could overcome these issues (92%). Human resource shortages were identified as being for doctors (74%), midwives (36%), nurses (30%), and specialists (6%).

#### Prevailing perceptions, attitudes, views and knowledge of telemedicine

Patients identified multiple benefits of telemedicine: time saving (88%; “It will save time”, “Faster”), cost effective (72%; “Cost saving”, “Reduces costs and time”), convenient (46%; “Access to a Doctor any time of the day”, “Readily available”), and efficient (27%; “Improved efficiency leading to faster diagnosis”, “It will enable health professionals to help many people in a short time”). Possible reasons why others might not use telemedicine were: fear of technology (40%), fear of the unknown (21%), lack of confidentiality (18%), and resistance to change (16%). Three employed individuals also identified a lack of a legal framework. The main challenges to the implementation of telemedicine were considered to be technical (76%) and financial (43%).

Network issues (84%), privacy and confidentiality (40%), and obtaining prescriptions (27%) were identified by individuals as their own perceived risks of using telemedicine. More than half (55%) felt that cultural issues such as existing health beliefs and traditional medicine practices within their community would have a negative impact on the introduction of telemedicine. Comments reflected a variety of issues (“Since many are not familiar with the use of the Internet, they may find it disturbing and not private”; “Many people will feel that they are not taken seriously looking at the issue of illiterate people and those who cannot use computers, as well as the issue of cost”; “It is almost culture that patients need to see a doctor physically, hence they may be reluctant to telemedicine”; and “Beliefs in traditional doctors and churches will affect telemedicine”). A few respondents believed that telemedicine would be suitable for only young populations (“It favours the youth mostly, because elders do not know how to use computers”) and others indicated that the adult populace would perceive telemedicine to be only appropriate to young generations (“It will be initially difficult for Botswana to accept change so it will be slow at first and increase as more millennials are born”). Despite these perceptions, other comments gave hope of acceptance (“I think people will accept telemedicine and forget about their traditional practice”, and “It can affect it in a bad way but with time people will learn the use of telemedicine for good health benefits rather than be stuck to beliefs”).

Provided with a definition of telemedicine to consider, patients generally ‘strongly agreed’ or ‘agreed’ that telemedicine would increase access to specialists (88%), assist with patient referral (89%), and relieve overcrowding of health facilities (through better referral, 92%). However, some felt that they may not like loss of the doctor/patient personal touch with telemedicine (40%) and might reject telemedicine because of being uncomfortable with possible security and confidentiality issues (43%).

#### Factors influencing implementation of telemedicine

Given a choice of possible impacts of telemedicine, patients generally considered the following of most importance to them: increasing staff retention at community facilities (81%), better local diagnosis and treatment without referral (84%), and saving lives (71%). More particularly they believed telemedicine would enhance efficiency and reduce costs through early and local diagnosis and treatment and easing of system pressures (each about 93%).

## Discussion

Fewer than half of the doctors (42%) had used telemedicine and only 10% of the nurses, whilst patients had little experience of telemedicine, but generally believed they would like to have it. These findings were despite HCP generally having access to and using computers both at work and at home, and over half of employed patients also having access to and using computers at work. There was a strong willingness to learn about and use telemedicine and to use telemedicine for clinical, and educational purposes. This was potentially tempered by factors such as cultural attitudes and preconceptions (e.g., existing health beliefs and traditional medicine practices) and the concern of some patients about reluctance to change, age, and privacy issues.

Both groups identified similar benefits for the use of telemedicine: improved access to specialists and health facilities, cost savings, convenience, and preventive healthcare, and also believed telemedicine would be able to help address some of the identified healthcare issues. Given that most patients believed little or no effort had been made to address their previously identified health issues, this presents an opportunity for TM interventions. Although financial, infrastructural and technical issues were identified as challenges to telemedicine, as in other developing countries, human resource, equipment shortages, funding were the prime concerns of both healthcare professionals and patients [31, 32].

Significant research has been performed on the knowledge, attitude, practice, and perceptions of both clinicians and patients regarding eHealth, telehealth, telemedicine, and mHealth in Middle Eastern, Asian, and African developing countries. Literature findings align well with those of this study regarding general level of knowledge, belief in telemedicine as a solution, and willingness to use telemedicine for both HCP and patients or the public.

For example, in Saudi Arabia nearly half (46%) of physicians had low knowledge of telemedicine, with just over half (53%) being unfamiliar with telemedicine tools and associated medical applications and technology [32]. That study also identified more than 90% perceived telemedicine as a viable option for providing medical care services to patients, and more than 90% of specialists agreed that telemedicine could save time and money. Although a 2015 study, in Iran a survey found most clinicians (96%) had “little knowledge about telemedicine” [33]. This may have since changed with a recent review indicating that progress, although slow, has been made in Middle-Eastern countries despite cultural, technological, regulatory and other challenges [34].

Within Asia findings are also similar. In Bangladesh a survey indicated one quarter of doctors had good knowledge of eHealth, half had average knowledge, and one quarter had poor knowledge, with the mobile phone as the most frequently used modality [34, 35]. A study from Pakistan noted 65% of physicians were already familiar with telemedicine services, and that most agreed that telemedicine would improve physician efficacy (75%) and that such services would improve access to healthcare (80%) [36]. More positively, a second Pakistani study showed 81% of physicians were aware of a definition of telemedicine, with 28% believing telemedicine could provide faster care, but with 43% believing it disrupted the doctor-patient relationship and led to privacy breaches, and almost all (91%) believing poverty and lack of education were the greatest barriers to telemedicine in the developing world [37].

In Africa, a Nigerian study found that of multiple indicators, healthcare professionals possessed good overall knowledge of ‘ehealthcare’ delivery (86%), although eHealth use was low [38]. Of the general population of Egypt, half had used a telemedicine tool (50.4%; even before the COVID-19 pandemic) and it was concluded most Egyptians “perceive the benefits of telemedicine positively and are willing to use it” [39]. Even with the advent COVID-19 and introduction of telemedicine services within the country, only 37% of Libyan healthcare workers reported high awareness of telemedicine, whether in the public or private sectors, and the study identified the need for telemedicine training and support of healthcare workers [40].

The present study did not specifically pursue educational issues, cultural issues, or legal, ethical, and regulatory issues, although some responses touched on these topics, indicating a level of awareness. Again, findings were similar to published insight, where the need for training, resolution of legal and standards issues, and consideration of cultural issues are identified as requiring attention [31, 32]. Highlighting the need for training, a cross-sectional study in Ethiopia, designed to assess the basic digital competency of healthcare providers at public health facilities, concluded they lacked problem solving, safety, and communication competencies in using digital devices [41]. Also, half (50%) of healthcare workers in an Egyptian study were aware of telehealth, with their knowledge and attitude scores increasing after attending an educational program, demonstrating the value of training [42].

This study provides insights and specific guidance on how to frame an evidence-based telemedicine-specific strategy for the country. Thus, the results highlight areas identified by HCPs and patients upon which to focus: Clinical, Educational and Administrative. Based upon responses, clinical solutions could include consultations in areas such as radiology, pathology, and surgery, although 36 areas of medicine were identified for possible application. Similarly, use of telemedicine for education of both HCP (e.g. clinical skill building; training to use telemedicine) and patients (e.g. health awareness; training to use telemedicine) was identified by both groups. Even applications for administrative uses were proposed (e.g. access to laboratory results). Whilst not comprehensive, or necessarily literature based, these suggestions do show both HCP and patients see the potential applications of telemedicine to address identified health issues, needs, and concerns.

### Limitations

The level and scope of activity required at a national level could not be achieved within this exploratory study setting, requiring the eHSDF process to be mimicked. Whilst the findings learnt through this process are anticipated to be valid, the desired depth and breadth of insight may not have been achieved. For example, the survey questions were not granular enough to probe specific issues, nor to differentiate and align specific challenges, concerns, or issues with specific telemedicine solutions. In future research, key informant interviews of patients and healthcare professionals may allow greater examination of such issues. Given these limitations, it is recommended that future research sample at a larger scale.

## Conclusions

In general, knowledge and awareness of telemedicine in developing countries are poor, although perceptions and willingness to use are higher. The good alignment between the findings of this study and published studies lends strength to the intended use of the data for developing an evidence-based telemedicine-specific strategy for Botswana, as an adjunct to the existing eHealth strategy. Furthermore, although detailed knowledge and insight amongst healthcare workers and patients may be lacking in Botswana, the general acceptance, willingness to use, and perspective that telemedicine is helpful bodes well for any attempt to introduce telemedicine more systematically in the future, guided by an evidence-based strategy.

## Supporting information

S1 File Final survey questionnaires have been uploaded as supplementary material. (PDF)

## Lists of abbreviations

HCP: Healthcare Providers
ICT: Information and Communications Technologies
eHSDF: eHealth Strategy Development Framework
TM: Telemedicine
