# Supplementary material for: Perceptions and attitudes of patients and healthcare workers towards the use of telemedicine in Botswana: An exploratory study
Source: PLoS One. 2023 Feb 16;18(2):e0281754. doi: 10.1371/journal.pone.0281754 (PMC9934446; doi:10.1371/journal.pone.0281754)
Supplement: S1 File — (PDF) [file pone.0281754.s001.pdf]

Please complete the following demographic information:

1. Do you understand and consent to the study? Yes ☐ No ☐

   What is your:

2. Gender: ☐ male ☐ female
3. Age (Years): ☐ 16-25 ☐ 26-30 ☐ 31-40 ☐ 41-50 ☐ 51-60 ☐ 61 and more
4. Indicate your academic qualifications (check all that apply):

   Diploma ☐

   Higher Diploma ☐

   Degree ☐

   Masters Degree ☐

   PhD. ☐

5. Please state the country in which you obtained the qualification? E.g. Botswana, Lesotho, South Africa, Ghana, Australia, UK, USA, Japan, New Zealand, etc.

   …………………………………………………………………………………………………………………

6. What is your current Profession/Occupation? Please write it down:

   ………………………………………………………………………………………………………………...

7. Please enter the institution name of your health facility:

   ………………………………………………………………………………………………………………...

8. Do you work for the Government/Public Sector, Parastatal, Private Sector or NGO? Please tick the appropriate sector.

   Government/Public Sector ☐ Parastatal ☐ Private Sector ☐ NGO ☐

9. What is your specialisation? State the number of years of experience.
10. How many years since first qualifying in your field?
11. Do you use a computer to do your work

    a At work? Yes ☐ No ☐

    b. At home? Yes ☐ No ☐

12. Are you connected to the Internet?

a. At work Yes ☐ No ☐

b. At home. Yes ☐ No ☐

13. The following questions ask about your level of awareness or understanding about telemedicine. Please tick the most applicable statement to you.

a) Have you ever heard about telemedicine' Yes ☐ No ☐

b) IF YES: Please describe what you understand telemedicine to be? Use the space provided.

……………………………………………………………………………………………………………………………
……………………………………………………………………………………………………………………………

c) Have you ever used telemedicine? Yes ☐ No ☐

d) How have you ever used telemedicine? (Check all that apply).

Referral ☐

Ask for second opinion ☐

Give second opinion ☐

Education ☐

Other – please explain ☐

e) How was telemedicine performed? (Check all that apply).

Mail ☐

Cellphone "app' ☐

e-Mail ☐

Videoconferencing ☐

Facsimile (Fax) machine ☐

Other – please specify ☐

…………………………………………………………………………………………………………………………..

f) From which sources did you get the information on telemedicine? (Check all that apply).

Colleagues ☐

TV ☐

Internet ☐

Specialist Journal ☐

Scientific Meetings and Workshops ☐

Other – please explain ☐

………………..…………………………………………………………………………………………………

14. Has the Botswana Ministry of Health introduced telemedicine into some hospitals?

Yes ☐ No ☐

15. If YES, please choose from this list the telemedicine services that are being introduced (Check all that apply)

Telesurgery ☐

Women cervical cancer screening ☐

Telepathology ☐

Oral medicine ☐

Telepsychiatry ☐

Teledermatology ☐

Teleradiology ☐

Other – please specify ☐

***Below is a brief definition of Telemedicine to assist you in answering the subsequent questions***.

Craig and Patterson (2005) define telemedicine in two ways;

1. Telemedicine is the delivery of health care and the exchange of health care information across distances. The prefix "tele" derives from the Greek for "at a distance"; hence more simply, telemedicine is medicine at a distance. As such it encompasses the whole range of medical activities including diagnosis, treatment and prevention of disease, continuing education of health care providers and consumers, and research and evaluation.
2. Telemedicine is defined as; "Rapid access to shared remote medical expertise by means of telecommunications and information technologies, no matter where the patient or the relevant information is located"

16. Now that you have a clear definition of what telemedicine is, would you agree or disagree with the following statements? Please tick the appropriate box:

a) Telemedicine may be a cost effective way of delivering health services to rural and remote patients.

| Strongly Agree | Agree | Disagree | Strongly Disagree |
|---|---|---|---|
| | | | |

b) Using telemedicine, public patients will have the opportunity to access health specialist services that are ordinarily only available in large city/district hospitals.

| Strongly Agree | Agree | Disagree | Strongly Disagree |
|---|---|---|---|
| | | | |

c) Patients may not like telemedicine since it makes them lose that doctor/patient personal touch that the traditional health delivery methods present to patients.

| Strongly Agree | Agree | Disagree | Strongly Disagree |
|---|---|---|---|
| | | | |

d) Patients are likely to reject this way of providing medical health services because they are not comfortable with the security and confidentiality of their information (medical records), during the time it is sent through the telecommunication lines.

| Strongly Agree | Agree | Disagree | Strongly Disagree |
|---|---|---|---|
| | | | |

e) Telemedicine second opinion/teleconsultation will assist doctors and nurses to determine whether patient referral is needed..

| | | | |
|---|---|---|---|
| Strongly Agree | Agree | Disagree | Strongly Disagree |

f) When telemedicine avoids physical transfer to referral hospital, the hospitals are relieved of the burden of ward congestion and transportation costs.

| | | | |
|---|---|---|---|
| Strongly Agree | Agree | Disagree | Strongly Disagree |

g) Using telemedicine, remotely placed doctors will have the opportunity to access specialist health services that are ordinarily only available in large city/district hospitals.

| | | | |
|---|---|---|---|
| Strongly Agree | Agree | Disagree | Strongly Disagree |

17. What will be your optimum level of preference in using the following? Tick one level of importance that is applicable to you in questions a) through d).

a) Health informatics (electronic patient records, surveillance, monitoring).

| | | | | |
|---|---|---|---|---|
| Most Important | Important | Moderately Important | Slightly Important | Not Important |

b) Telehealth (interacting with other health care providers, ill patients, well citizens e.g. teleconsultations, social networking).

| | | | | |
|---|---|---|---|---|
| Most Important | Important | Moderately Important | Slightly Important | Not Important |

c) e-learning (e.g. providing teaching and education opportunities for healthcare providers and patients).

| | | | | |
|---|---|---|---|---|
| Most Important | Important | Moderately Important | Slightly Important | Not Important |

d) e-commerce (related to business process for health care, e.g. electronic reimbursements)

| Most Important | Important | Moderately Important | Slightly Important | Least Important |
|---|---|---|---|---|
| | | | | |

18. What are the most pressing health related problems/challenges or needs in your working environment? These needs should only be focused on and related to healthcare, health services, or health-related information. (a, b, c)

.......................................................................................................................................................
.......................................................................................................................................................
.......................................................................................................................................................
...... .................................................................................................................................................

19. Of the diseases that you encounter in your working environment, which ones do you consider to be the top five and that need immediate attention? List the diseases in order of importance, with the most important listed first.

1 ……………………………………………………..

2.…………………………………………………….

3.…………………………………………………….

4.…………………………………………………….

5.…………………………………………………….

20. In your opinion do you think there is a shortage of resources in the health sector in your community - whether concerning human, material or technological resources?

a). Human Health Resources (e.g., midwives, nurses, physicians)? Yes ☐ No ☐

b). Material Resources (e.g., remote clinics; hospital beds; medical equipment)? Yes ☐ No ☐

c). Technological Resources (e.g., connectivity; telemedicine equipment)? Yes ☐ No ☐

d). Financial Resources (e.g., budgets)? Yes ☐ No ☐

21. Do you believe that telemedicine can be introduced to overcome and address the health needs and health shortages you identified in Q19 and Q 20? Yes ☐ No ☐

22. If human health resources shortages exist, in which particular cadres (midwifes, doctors, nurses, etc.) do they exist? (a, b, c)

23. Does your health facility use telemedicine technologies? Such as videoconferencing, remote education, etc. Yes ☐ No. ☐

24. If your hospital DOES use telemedicine, what does it use it for? Tick all that apply.

    Clinical Consultations ☐

    Education ☐

    Meetings ☐

    Other – please explain ☐

25. Which telemedicine technologies are used in your health facility? (Check all that apply)

    Telephone call ☐

    Cellphone "app" ☐

    e-Mail ☐

    Videoconferencing ☐

    Facsimile (Fax) machine ☐

    Other – please explain ☐

    ……………………………………………………………………………………………………

26. If you have never used telemedicine before, do you think members of the health community would be willing and open to using it? Yes ☐ No ☐

27. What would be the three main reasons for using telemedicine in your work environment?

    1) ……………………………………………………………………………………………………………
    2) ……………………………………………………………………………………………………………
    3) ……………………………………………………………………………………………………………

28. For what health services would you prefer to use telemedicine? (a,b,c,d,e)

    ……………………………………………………………………………………………………………
    ……………………………………………………………………………………………………………
    ……………………………………………………………………………………………………………
    ……………………………………………………………………………………………………………
    ……………………………………………………………………………………………………………

29. Should you consider using telemedicine, what would be the main challenges and obstacles that will be encountered by your community? a) You, b) Other Providers, c)Patients,
…………………………………………………………………………………………………………………
…………………………………………………………………………………………………………………
………………………………………………………………………………………………………………

30. Do you believe that your community will be in a position to address these challenges and obstacles?

    a. You Yes ☐ No ☐

    b. Other Providers Yes ☐ No ☐

    c. Patients Yes ☐ No ☐

31. Do you think there will be other alternative options to telemedicine that would be more appropriate to address the health needs of your community? Yes ☐ No ☐

32. Does the Botswana Ministry of Health have a health strategy? Yes ☐ No ☐ Do Not Know

33. If you would like to be considered for further participation within focus group discussions, please provide your e-mail address?

…………………………………………………………………………………………………………………..

34. I hereby confirm my availability to participate in the focus group. Yes ☐ No ☐

Nelson R Mandela School of Medicine

College of Health Science

University of KwaZulu-Natal

Durban Campus

**INFORMED CONSENT LETTER**

## - Invitation to Participate in a Research Survey -

## Development of a telemedicine strategy for Botswana by Implementing the 'e-Health Strategy Development Framework'

Dear Participant

You are cordially invited to participate in a research study titled "Development of a telemedicine strategy for Botswana by implementing the e-Health Strategy Development Framework". This study is being conducted by Benson Ncube (Principal Researcher) and his research supervisors from the Department of Telehealth at the University of KwaZulu-Natal. The purpose of this study is to;

- identify and prioritize unmet e-health needs within Botswana
- determine optimal, cost effective and appropriate telemedicine initiatives
- determine the communities that need more health services
- develop a telemedicine strategy for Botswana

In this study, you will be asked to complete an interview questionnaire that will bring forth your views, perceptions, attitudes and opinions on telemedicine developments. Your participation in this study is voluntary and you are free to withdraw your participation from this study at any time without any obligation. The survey should take only 15 minutes to complete.

This survey has been approved by the Institutional Review Board of University of KwaZulu-Natal as well as Botswana Ministry of Health & Wellness Research Ethics Committee. There are no risks associated with participating in this study. The survey collects no identifying information of any respondent. All of the responses to the survey will be recorded anonymously. The reporting on the research results will be aggregated, and therefore it will be impossible to link the results with any research participant.

While you will not experience any direct benefits from participation, information collected in this study may benefit medical practitioners and improve future patient outcomes, e.g., critical resources within the Ministry of Health &

Wellness may be shared more equitably through the use of telemedicine, and appropriate telemedicine initiatives may be implemented where they are needed most. Ultimately the strategy will assist the government in achieving the WHO targets to improve the welfare of Batswana.

Thanking you for your assistance in this research process.

Yours sincerely

Benson Ncube

Contacts: E-Mail: bns@btcmail.co.bw and Mobile +267 73192352

Research Office Contact: Mr. Prem Mohun, HSSREC Research Office, Tel +27 312604557;

Fax +27 312604609, E-Mail: mohunp@ukzn.ac.za

**DECLARATION OF CONSENT**

I ……………………………………………………………………………… have read the above information and I understand it. I therefore, hereby declare that I have consented to be part of this research study.

Signature ……………………………………………………… Date ………………………………………………………..

Please complete the following demographic information:

What is your:

1. Gender: ☐ male ☐ female
2. Age (Years): ☐ 16-25 ☐ 26-30 ☐ 31-40 ☐ 41-50 ☐ 51-60 ☐ 61 and more
3. Write down your current Profession/Occupation. …………………………………………………………………….
4. Which health facilities do you usually use? Please tick the appropriate sector.

   General Hospital ☐ Government Clinic ☐ Private GP ☐ Private Hospital ☐ Other ☐

   Other (describe) ……………………………………………………………………………………………………

5. Do you use a computer to do your work? Yes ☐ No ☐
6. Are you connected to the Internet? Check one in question 6a and question 6b.
   a. At work Yes ☐ No ☐
   b. At home. Yes ☐ No ☐

***Below is a brief definition of Telemedicine to assist you in answering the subsequent questions.***

Craig and Patterson (2005) define telemedicine in two ways;

3. Telemedicine is the delivery of health care and the exchange of health care information across distances. The prefix "tele" derives from the Greek for "at a distance"; hence more simply, telemedicine is medicine at a distance. As such it encompasses the whole range of medical activities including diagnosis, treatment and prevention of disease, continuing education of health care providers and consumers, and research and evaluation
4. Telemedicine is defined as; "Rapid access to shared remote medical expertise by means of telecommunications and information technologies, no matter where the patient or the relevant information is located"

7. From which sources did you get the information on telemedicine? (Check all that apply).

   Word of Mouth ☐

TV ☐

Internet ☐

Government Speech ☐

Health Meetings and Workshops ☐

Other (describe) …………………………………………………………………………………………………

8. Did you know that the Botswana Ministry of Health has now introduced telemedicine in some hospitals?
Yes ☐ No ☐

9. If NO, are you willing to learn about and use telemedicine?

Yes ☐ No ☐

10. Now that you have a clear explanation of what telemedicine is, how much would you agree or disagree with the following statements? Please circle the word / phrase that best applies to you.

a) Using telemedicine, we as patients will have the opportunity to access specialist health services that are ordinarily only available in large city/district hospitals.

| | | | |
|---|---|---|---|
| Strongly Agree | Agree | Disagree | Strongly Disagree |

b) We as patients may not like telemedicine since it makes us lose that doctor/patient personal touch that the traditional health delivery methods provide.

| | | | |
|---|---|---|---|
| Strongly Agree | Agree | Disagree | Strongly Disagree |

c) We as patients are likely to reject this way of providing health services, because we are not comfortable with the security and confidentiality of our information (medical records), at the time information is sent through the telecommunication lines.

| | | | |
|---|---|---|---|
| Strongly Agree | Agree | Disagree | Strongly Disagree |

d) Telemedicine will assist the doctor/nurse to screen us properly for referring us as patients to referral hospitals.

| | | | |
|---|---|---|---|
| Strongly Agree | Agree | Disagree | Strongly Disagree |

e) When patients are properly referred, the referral hospitals will be relieved of overcrowding of ward rooms and ambulance services (transportation costs).

| | | | |
|---|---|---|---|
| Strongly Agree | Agree | Disagree | Strongly Disagree |

f) If telemedicine was to be available in my community hospital, I would definitely use it.

| Strongly Agree | Agree | Disagree | Strongly Disagree |
|---|---|---|---|
| | | | |

***The following statements discuss the importance of telemedicine to both the community and the Government.***

11. Please indicate from your own point of view the importance of the following statements.

a) Telemedicine could contribute as a way to keep medical professionals working in remote community hospitals

| Most Important | Important | Moderately Important | Slightly Important | Not Important |
|---|---|---|---|---|
| | | | | |

b) When using telemedicine, diagnosis and treatment would be possible without physically transporting the patient to the referral hospital.

| Most Important | Important | Moderately Important | Slightly Important | Not Important |
|---|---|---|---|---|
| | | | | |

c) More lives are likely to be saved when telemedicine is properly used.

| Most Important | Important | Moderately Important | Slightly Important | Not Important |
|---|---|---|---|---|
| | | | | |

d) Telemedicine has the potential to reduce costs by facilitating early diagnosis and treatment at the local community hospital

| Most Important | Important | Moderately Important | Slightly Important | Least Important |
|---|---|---|---|---|
| | | | | |

e) Early treatment results in freeing hospital wards and ambulance space leading to reduction in associated costs

| Most Important | Important | Moderately Important | Slightly Important | Least Important |
|---|---|---|---|---|
| | | | | |

f) Early treatment results in freeing scarce health facilities and specialists to provide service to those patients who critically need their interventions

| Most Important | Important | Moderately Important | Slightly Important | Least Important |
|---|---|---|---|---|
| | | | | |

12. What are the most pressing health related problems/challenges or needs in your community? These needs should only be focused on and related to healthcare and health services.

..........................................................................................................................................................
..........................................................................................................................................................
..........................................................................................................................................................
......

13. List the top five diseases that affect your community. List them in order of the priority of treating such diseases.

    1 ……………………………………………………..

    2.……………………………………………………..

    3.……………………………………………………..

    4.……………………………………………………..

    5.………………………………………………………

14. In your opinion do you think there is a shortage of resources in the health sector in your community - whether concerning human, material or technological resources?

    a). Human Health Resources (e.g., midwives, nurses, physicians)? Yes ☐ No ☐

    b). Material Resources (e.g., remote clinics; hospital beds; medical equipment)? Yes ☐ No ☐

    c). Technological Resources (e.g., connectivity; telemedicine equipment)? Yes ☐ No ☐

15. Do you believe that telemedicine can be introduced to overcome and address the health needs and shortages you identified in questions 12 and 14?

    Yes ☐ No ☐

16. If human resources shortages exist, in which particular health specialties (midwifes, doctors, nurses, etc.) do they exist?

..........................................................................................................................................................
..........................................................................................................................................................

17. In the past what measures, if any, have been taken to resolve these problems within your community?

……………………………………………………………………………………………………………………
……………………………………………………………………………………………………………………
……………………………………………………………………………………………………………………

18. Select your referral hospital from the list given below.

Princess Marina Hospital ☐

Nyangabgwe Hospital ☐

Lobatse Hospital ☐

Scottish Livingstone Hospital ☐

Bamalete Hospital ☐

Athlone Hospital ☐

19. How much time do you need to travel from where you live to the nearest referral hospital that offers better health services? Please tick the appropriate time period.

Up to 2 hours ☐ 2-4 hours ☐ 4-6 hours ☐ 6-8 hours ☐ More than 8 hours ☐

20. The time taken and conditions of travel to the referral hospital can have severe and disastrous consequences for those in need. To what extent do you agree or disagree with this statement? Check one.

| Strongly Agree | Agree | Disagree | Strongly Disagree |
|---|---|---|---|

21. If it were possible, I would access the health facilities remotely from my community hospital or clinic using Telemedicine. To what extent do you agree or disagree with this statement? Check one.

| Strongly Agree | Agree | Disagree | Strongly Disagree |
|---|---|---|---|

22. Consulting a medical doctor using telephone lines or Internet will be cheaper for me and for the Government. To what extent do you agree or disagree with this statement? Check one.

| Strongly Agree | Agree | Disagree | Strongly Disagree |
|---|---|---|---|

23. Although it will be faster and cheaper to send my information to a remote hospital, I would prefer to travel and wait for long hours to see the doctor on a face-to-face basis. To what extent do you agree or disagree with this statement? Check one.

| Strongly Agree | Agree | Disagree | Strongly Disagree |
|---|---|---|---|

24. I consider it uncultured and inhumane to have one being diagnosed and treated through the Internet by a remotely located doctor. To what extent do you agree or disagree with this statement? Check one.

| Strongly Agree | Agree | Disagree | Strongly Disagree |
|---|---|---|---|

25. If I am properly trained and skilled to use a computer to consult a remote doctor and I am given the assurance that the doctor is legally licensed to practice medicine in this manner, I would confidently use the service. To what extent do you agree or disagree with this statement? Check one.

| Strongly Agree | Agree | Disagree | Strongly Disagree |
|---|---|---|---|

26. I will encourage other persons in the community to use telemedicine because telemedicine will promote access to shared critical national resources in an equitable way to include the underprivileged and remote communities. To what extent do you agree or disagree with this statement? Check one.

| Strongly Agree | Agree | Disagree | Strongly Disagree |
|---|---|---|---|

27. What do you think might cause most people to avoid using telemedicine? Please check ALL that apply.

| Fear of technology | Lack of confidentiality | Resistance to change | Fear of the unknown | Lack of legal framework | None |
|---|---|---|---|---|---|

28. In this modern time many people are computer literate and spend most of their leisure time on social networks, such as Twitter, Skype, LinkedIn, WhatsApp, Facebook, Google, etc. To what extent do you agree or disagree with this statement? Check one.

| Strongly Agree | Agree | Disagree | Strongly Disagree |
|---|---|---|---|

29. If people are very comfortable when using social media, they should also be comfortable to use telemedicine that will improve their treatment and health outcomes. To what extent would you agree or disagree with this statement? Check one.

| Strongly Agree | Agree | Disagree | Strongly Disagree |
|---|---|---|---|
| | | | |

30. In situations whereby a surgical operation may need to be performed, I will always go to (choose one)::

    Princess Marina Hospital ☐

    Gaborone Private Hospital ☐

    Bokamoso Hospital ☐

    Nyangabwe Hospital ☐

    State-referred Hospital ☐

    Other ☐

    If other please specify
    ……………………………………………………………………………………………………………

31. In your own view what do you consider to be the three main advantages or benefits related to using telemedicine?
    1. ……………………………………………………………………………………………………………
    2. ……………………………………………………………………………………………………………
    3. ……………………………………………………………………………………………………………

32. Should you consider using telemedicine, what do you think would be the main challenges and obstacles that will be encountered by your community? Check those that would apply to your community

    Financial limitations ☐

    Lack of electricity ☐

    Patients' resistance to change ☐

    Lack of telecommunications infrastructure ☐

    Skills limitations in the area of telemedicine ☐

    Changes in Government development priorities ☐

33. In your own view what would you consider to be the risks associated with introducing telemedicine? List the top three risks.

    1. ……………………………………………………………………………………………………………

2. ………………………………………………………………………………………………………………
3. ………………………………………………………………………………………………………………

34. How will the existing health beliefs and traditional medicine practices within your community affect the introduction of telemedicine as a means of providing health services?
………………………………………………………………………………………………………………………
………………………………………………………………………………………………………………………
………………………………………………………………………………………………………………………

35. Do you think the public should have a stake in the making of Botswana Health Policy? Yes ☐ No ☐
Please give at least one reason why:
………………………………………………………………………………………………………………………
………………………………………………………………………………………………………………………

36. Have you ever visited the website of the Ministry of Health in Botswana? Yes ☐ No ☐

If YES;

Does it facilitate citizen interaction? Yes ☐ No ☐

Is it helpful and informative? Yes ☐ No ☐

Why did you visit the website?……………………………………………………………………………………..
